# *Sphaerosyllis levantina* sp. n. (Annelida) from the eastern Mediterranean, with notes on character variation in *Sphaerosyllis hystrix* Claparède, 1863

**DOI:** 10.3897/zookeys.150.1877

**Published:** 2011-11-28

**Authors:** Sarah Faulwetter, Georgios Chatzigeorgiou, Bella S. Galil, Artemis Nicolaidou, Christos Arvanitidis

**Affiliations:** 1 Department of Zoology-Marine Biology, Faculty of Biology, National and Kapodestrian University of Athens, Panepistimiopolis, 15784, Athens, Greece; 2 Department of Biology, University of Crete, 71409 Heraklion, Crete, Greece; 3 National Institute of Oceanography, Israel Oceanographic & Limnological Research, POB 8030, Haifa 31080, Israel; 4 Institute of Marine Biology and Genetics, Hellenic Centre for Marine Research, 71003 Heraklion, Crete, Greece

**Keywords:** Polychaetes, Syllidae, Exogoninae, *Sphaerosyllis*, new species, Mediterranean, Cybertaxonomy, Scratchpads

## Abstract

Examination of polychaete specimens from Haifa Bay (Israel, eastern Mediterranean Sea) revealed several individuals exhibiting morphological characteristics similar to *Sphaerosyllis hystrix* Claparède, 1863. A detailed morphometrical analysis of the Israeli specimens in comparison to specimens of *Sphaerosyllis hystrix* and *Sphaerosyllis boeroi* Musco, Çinar and Giangrande, 2005 supported the description of the former as a new species, *Sphaerosyllis levantina* **sp. n.** Individuals of *Sphaerosyllis hystrix* formed a very heterogeneous group with strong character variations in the analysis and the presumed cosmopolitan distribution of the species is discussed based on literature records.

## Introduction

The polychaete genus *Sphaerosyllis* Claparède, 1863 (Annelida) is one of the most species-rich genera of the syllid subfamily Exogoninae. At present, ca. 48 species are considered valid within *Sphaerosyllis* after the recent split of the group into the three genera *Sphaerosyllis*, *Prosphaerosyllis* and *Erinaceusyllis* (San Martín 2005). Up to date, 18 species of the genus have been recorded from the Mediterranean Sea (Musco and Giangrande 2005), one of them described but yet unnamed (San Martín 2003), another one in the process of description (Del Pilar-Ruso and San Martín in press). In the framework of a project focusing on the soft bottom benthos of Haifa Bay (Israel, eastern Mediterranean Sea), a number of individuals of the genus *Sphaerosyllis* were found to exhibit morphological features which did not entirely correspond to any description of known *Sphaerosyllis* species, namely falcigers with a strong serration and with a subdistal spine present in all chaetigers. A subdistal spine on the blades of at least some falcigers has been described for the type species of the genus, *Sphaerosyllis hystrix* Claparède 1863, and for *Sphaerosyllis boeroi* Musco, Çinar and Giangrande, 2005. Re-examination of material of *Sphaerosyllis hystrix* revealed that some individuals –contrary to descriptions available in the literature– possess a subdistal spine not only on the blades of the falcigers in anterior but also in posterior chaetigers. Consequently, this characteristic could not be used to unambiguously distinguish the Israeli material from *Sphaerosyllis hystrix*. In order to clarify the relationship between the three very similar species possessing falcigers with a subdistal spine, a morphometric analysis has been performed, a method allowing not only to discriminate statistically significant groupings but also to identify taxonomically important characters (Costa-Paiva and Paiva 2007).

## Material and methods

### Specimen collection and processing

Specimens were collected on 11 Oct. 2009 in Haifa Bay, (Israel, Eastern Mediterranean Sea) from fine to medium sands in shallow waters (10 m). Sediment samples were taken with a Van-Veen grab (KAHLSICO, model WA265/SS214) 32×35 cm, volume 20 l, penetration 20 cm. The sediment was preserved in buffered formalin 10% for 3–7 days, then sieved through a 250 µm mesh sieve and subsequently stored in 70% ethanol. Specimens were examined under an Olympus SZx12 stereomicroscope and an Olympus BX50 microscope. Illustrations in pencil were made by means of a drawing tube, subsequently scanned, imported into a graphic program (GIMP), re-drawn and saved as a vector graphic. Three specimens selected for obtaining Scanning Electron Microscope (SEM) images were dehydrated, critical point dried (Bal-Tec CPD 030), sputter-coated with gold (Bal-Tec SCD 050) and examined under a JEOL JSM-6390LV at the Department of Biology, University of Crete. Specimens are deposited in the invertebrate collection of the Smithsonian National Museum of Natural History, Washington D.C., USA (USNM) and in the Tel Aviv University Zoological Museum, Israel (TAU).

### Morphometric analyses

A total of 30 individuals belonging to three species (*Sphaerosyllis boeroi*: 3 individuals; *Sphaerosyllis hystrix*: 21 individuals; *Sphaerosyllis levantina* sp. n.: 6 individuals) were analysed. Twenty-five variables were measured: I. body length, to account for size-dependencies of other characters; II. number of chaetigers; III. length of blade of dorsalmost falciger of a) anterior, b) midbody, c) posterior chaetigers; IV. length of blade of ventralmost falciger of a) anterior, b) midbody, c) posterior chaetigers; V. ratio of length of blades of dorsalmost to ventralmost falciger in a) anterior, b) midbody, c) posterior chaetigers; VI. ratio of length of blades of falcigers in anterior to posterior chaetigers for a) dorsalmost; b) ventralmost falciger; VII. Ratio of length of dorsalmost falciger to body length in in a) anterior, b) midbody, c) posterior chaetigers; VIII. Ratio of length of ventralmost falciger to body length in in a) anterior, b) midbody, c) posterior chaetigers; IX. maximum length of serration of falcigerous blades in a) anterior, b) midbody, c) posterior chaetigers (smooth, finely serrated, strongly serrated); X. presence of a subdistal spine in dorsalmost falcigerous blades of in a) anterior, b) midbody, c) posterior chaetigers.

Body length was measured excluding antennae, anal cirri and palps. Falciger blade lengths were measured from point of insertion into shaft to distal tip. Falciger blade lengths could not always be measured on the same chaetiger in all animals if blades were broken. Instead, measurements were made in predefined body regions (anterior: first 1–5 chaetigers; posterior: last 5–7 chaetigers; midbody: in between). Three individuals of *Sphaerosyllis hystrix* were excluded from the multivariate statistical analysis due to missing values for some characters.

Summary statistics (mean, minimum, maximum, standard deviation, coefficient of variation and range of values) were calculated for each species (measurements and calculations available in online supplementary material:

http://polychaetes.marbigen.org/content/measured-values-sphaerosyllis-specimens

http://polychaetes.marbigen.org/content/summary-statistics-sphaerosyllis-hystrix

http://polychaetes.marbigen.org/content/summary-statistics-sphaerosyllis-boeroi

http://polychaetes.marbigen.org/content/summary-statistics-sphaerosyllis-levantina)

To take the different data types (numerical, categorical, binary) into account, Gower’s similarity coefficient (Gower 1971) was chosen to calculate a similarity matrix. Multidimensional Scaling (MDS) was subsequently employed to display the similarities of the different individuals. To test for significance of differences between species a PERMANOVA (Permutational Multivariate Analysis of Variance) was performed (Anderson 2001). A Principal Component Analysis (PCA) was used to determine variability of characters and to identify characters for the species differentiation. To determine the importance of the characters discriminating the species, the Principal Component Scores were correlated (Spearman’s correlation coefficient) with the measured character values of each individual.

Multivariate statistical analyses were performed with PRIMER V6, correlation of the Principal Component Scores were calculated with the R package (R package version 2.10; http://www.R-project.org).

### Electronic publication

The description of the new taxon was prepared in a Virtual Research Environment (Scratchpads) allowing for rapid and simultaneous publication of the results in print as well as electronically in a semantically enhanced form (Blagoderov et al. 2010, Penev et al. 2010). This publication and all supplementary data (measurements, results of statistical analyses, images) can be accessed on the Polychaete Scratchpads (http://polychaetes.marbigen.org).

## Results

### Taxonomic results

#### Sphaerosyllis levantina sp. n.

urn:lsid:zoobank.org:act:9CEE8F90-9596-49F6-AA22-BB79C0E816D9

http://species-id.net/wiki/Sphaerosyllis_levantina

**Figure 1.**
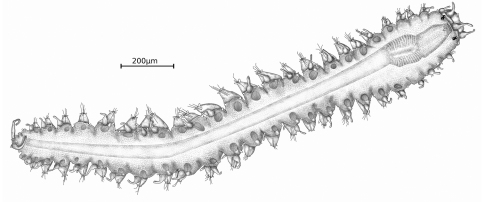
*Sphaerosyllis levantina* sp. n. holotype, dorsal view

Figures 1 [Fig F2] [Fig F3] 4

##### Type material.

Holotype (USNM 1160540) ALA-IL-7, Haifa Bay, 10.5 m depth. Label: “*Sphaerosyllis levantina*, Haifa Bay, coll. B. Galil 11.10.09 [Holotype]”. Paratypes USNM 1160541–1160573: 33 individuals, TAU-AN 25006: 10 individuals; Haifa Bay, Israel, Eastern Mediterranean Sea, Station ALA-IL-7, coll. 11.10.2009, depth 10.5 m; Labels: “*Sphaerosyllis levantina*, Haifa Bay, coll. B. Galil 11.10.09 [Paratype X]” (where X=1–43). All material preserved in 96% Ethanol.

##### Comparative material examined

**.** *Sphaerosyllis boeroi* Musco, Çinar, and Giangrande, 2005 (Southern Evoikos Gulf, Aegean Sea, Greece: 3 specimens [Label: Tribe *Sphaerosyllis*]). *Sphaerosyllis hystrix* (Southern Evoikos Gulf, Aegean Sea, Greece: 1 specimen [Label: Tribe *Sphaerosyllis*]; Northern Evoikos Gulf, Aegean Sea, Greece: 7 specimens [Label: DI9a 7.3.91 *Sphaerosyllis hystrix*, checked S.Martín], all deposited the in Hellenic Centre for Marine Research, Anavyssos, Greece; Chalkida, Aegean Sea, Greece: 1 specimen [Label: 56 – *Sphaerosyllis hystrix*, κατώτερη µεσοπαραλιακή Χαλκίδας, Στενά Ευρίπου, Ξενοδοχείο Λούσι, St. 18, 25.9.97 0-0.5m, Άτοµα: 1, Διδακτορικού Mίλτου] (= lower intertidal zone, Chalkida, Eviros Straight, Hotel Lousi, coll. M.S. Kitsos), Chalkida, Aegean Sea, Greece: 1 specimen [Label: 26 – *Sphaerosyllis hystrix*, κατώτερη µεσοπαραλιακή Χαλκίδας, Στενά Ευρίπου, Ξενοδοχείο Παλίρροια, St. 1a, 24.9.97 0-0.5m, Άτοµα: 1, Διδακτορικού Mίλτου] (= lower intertidal zone, Chalkida, Eviros Straight, Hotel Palirroia, coll. M.S. Kitsos), Chalkida, Aegean Sea, Greece: 6 specimens [Label: 33 – S*phaerosyllis hystrix*, κατώτερη µεσοπαραλιακή Χαλκίδας, Στενά Ευρίπου, Ξενοδοχείο Παλίρροια, St. 1α, 24.9.97 0-0.5m, Άτοµα: 6, Διδακτορικού Mίλτου] (= lower intertidal zone, Chalkida, Eviros Straight, Hotel Palirroia, coll. M.S. Kitsos), Chalkida, Aegean Sea, Greece: 4 specimens [Label: 80 – *Sphaerosyllis hystrix*, κατώτερη µεσοπαραλιακή Χαλκίδας, Στενά Ευρίπου, Ξενοδοχείο Παλίρροια, St. 1α, 24.9.97 0-0.5m, Άτοµα: 6, Διδακτορικού Mίλτου] (= lower intertidal zone, Chalkida, Eviros Straight, Hotel Palirroia, coll. M.S. Kitsos), Thessaloniki, Aegean Sea, Greece, 1 specimen [Label: 66 – *Sphaerosyllis hystrix*, κατώτερη µεσοπαραλιακή Λιµάνι Θεσσαλονίκης, 2γ, 6.10.97 0-0.5m, Άτοµα: 1, Διδακτορικού Mίλτου]) (= lower intertidal zone, Port of Thessaloniki, coll. M.S. Kitsos), all deposited the in Zoological Museum of the Aristotle University of Thessaloniki, Greece.

##### Type locality.

Eastern Mediterranean Sea, Levantine Basin, Israel, Haifa Bay (32°54.533N, 35°04.071E).

##### Description.

**Figure 2. F2:**
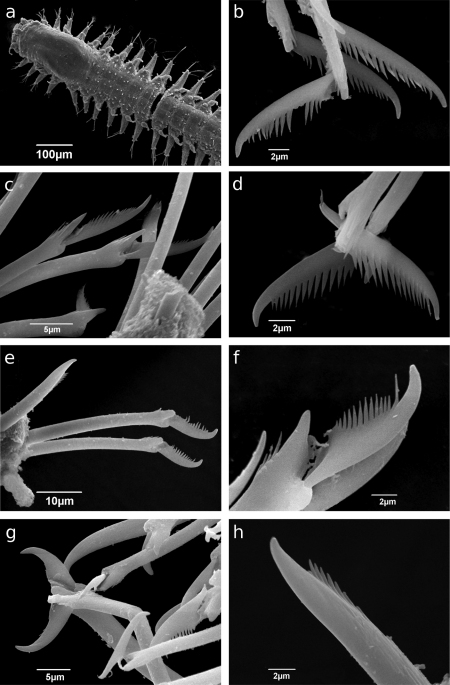
*Sphaerosyllis levantina* sp. n. SEM images of **a** anterior end and midbody, dorsal view **b–c** compound chaetae, anterior chaetigers **d** dorsalmost compound chaetae, anterior chaetiger **e** compound and dorsal simple chaetae, midbody **f** dorsalmost compound chaeta, posterior chaetiger **g** ventralmost compound chaetae, posterior chaetiger **h** dorsal simple chaeta

**Figure 3. F3:**
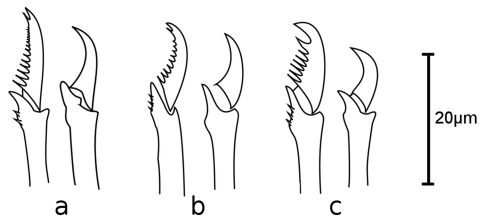
*Sphaerosyllis levantina* sp. n. Dorsal (left) and ventral (right) falciger of **a** anterior **b** midbody **c** posterior chaetiger

**Figure 4.**
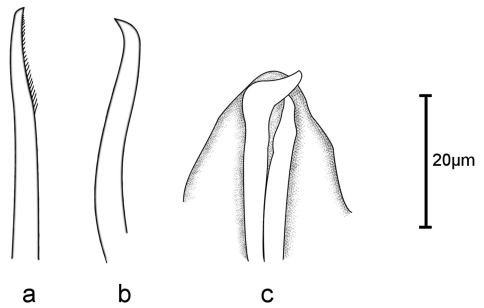
*Sphaerosyllis levantina* sp. n. **a** dorsal **b** ventral simple chaeta **c** aciculae, anterior chaetiger

Holotype, entire animal, with 25 chaetigers, length 1.9 mm with palps but without anal cirri; width at sixth chaetiger 250 µm without parapodia, 300 µm with parapodia. Body small, slender, widest at level of proventricle (Fig. 1). Dorsal papillation on anterior chaetigers irregular, after proventricle in four longitudinal rows: two mid-dorsal rows with two papillae per segment, lateral rows with three papillae near dorsal cirri (Fig. 2a). Ventrum without visible papillation. Prostomium wider than long with 4 coalescent lensed eyes in trapezoidal arrangement. Anterior eyespots absent. Antennae pyriform with bulbous bases and elongated tips, median antenna 40 µm long, lateral ones 33 µm, longer than prostomium and palps together. Median antenna inserted between anterior pair of eyes, lateral ones attached on anterior margin of prostomium (Fig. 1). Palps directed ventrally, fused along their length, with a dorsal notch and few small papillae. Peristomium indistinct, dorsal fold partly covering prostomium. One pair of tentacular cirri, shaped like antennae but shorter (23 µm). Second chaetiger without dorsal cirri but with large papilla instead. Dorsal cirri similar in shape and length to tentacular cirri, anteriorly as long as parapodial lobes (23 µm), posteriorly slightly longer (28 µm). Ventral cirri conical, half as long as parapodial lobe, originating at bases of parapodia. Parapodial lobes triangular, with small papilla on each side of distal end. Parapodial glands with fibrillar material and with conical opening; from fourth chaetiger. Anterior parapodia with 4–5, rarely with 6 falcigers per fascicle; blades slender, unidentate with small subdistal spine and strong serration on 1–2 dorsalmost falcigers (Figs 2b–d, 3a). Dorso-ventral gradation in length of blades, dorsal ones maximally 14 µm, ventral ones 10 µm. Posteriorly, dorsal blades of similar length (13 µm), but stouter and more curved with robust subdistal spine and strong serration as long as subdistal spine (Figs 2e, f, 3b, c). Dorsalmost falciger posteriorly thicker than remaining ones in fascicle. Blades of ventral falcigers similar throughout body (Fig. 2g). All shafts with fine serration (Fig. 2c). Dorsal simple chaeta from chaetiger 1, subdistally serrated (Figs 2h, 4a). Ventral simple chaeta on posterior chaetigers, sigmoid, smooth (Fig. 4b). Anteriorly two aciculae per parapodium, one distally bent at right angle, acuminate tip curved upwards, the other straight and blunt (Fig. 4c); posteriorly only one acicula of the former type per parapodium. Pharynx occupying three chaetigers. Width more than ¾ of width of proventricle. Pharyngeal tooth located on anterior margin, surrounded by a crown of soft papillae. Proventricle in chaetigers 3–4 (120 µm long) with 15–17 muscle cell rows. Pygidium papillated, with two cirriform anal cirri twice as long as dorsal cirri (60 µm) (Fig. 1).

##### Etymology.

Derived from the type locality (Levantine Basin), levantina being a neo-Latin adjective meaning “pertaining to the region where the sun raises”; feminine declination in accordance with the genus name (Syllis was a river nymph in the greek mythology and thus female).

##### Distribution.

Israeli Coast (Levantine Basin, Eastern Mediterranean Sea).

##### Habitat.

Fine to medium sands.

##### Taxonomic remarks.

**Figure 5.**
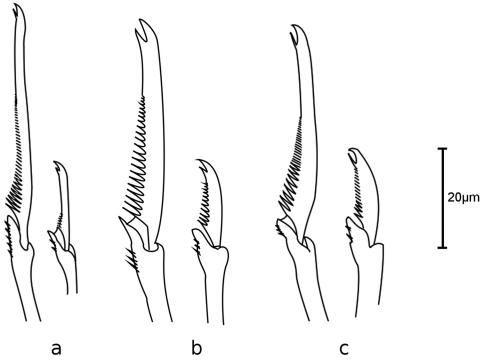
*Sphaerosyllis boeroi*. Dorsal (left) and ventral (right) falciger of **a** anterior **b** midbody **c** posterior chaetiger

**Figure 6.**
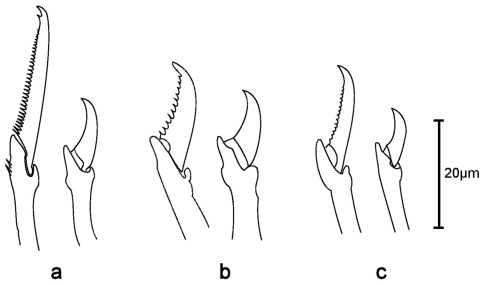
*Sphaerosyllis hystrix*. Dorsal (left) and ventral (right) falciger of **a** anterior **b** midbody **c** posterior chaetiger

*Sphaerosyllis levantina* sp. n. is similar to *Sphaerosyllis minima* Hartmann-Schröder, 1960 in having blades of falcigers with strong serration throughout the body. However, *Sphaerosyllis minima* has a stronger dorso-ventral gradation of the blades of falcigers (dorsal ones twice as long as ventral ones) than *Sphaerosyllis levantina* sp. n. (dorsal ones 1.5 times longer than ventral ones) and the ventral cirrus is longer than the parapodial lobe in *Sphaerosyllis minima*, whereas is is half as long as the parapodial lobe in *Sphaerosyllis levantina* sp. n. *Sphaerosyllis capensis* Day, 1953, *Sphaerosyllis taylori* Perkins, 1981, and *Sphaerosyllis sandrae* Álvarez and San Martín, 2009 are similar to *Sphaerosyllis levantina* sp. n. in the shape and serration of the blades of the falcigers, but *Sphaerosyllis capensis* has all antennae positioned in line (median one posteriorly of lateral ones in *Sphaerosyllis levantina* sp. n.), *Sphaerosyllis taylori* shows no dorso-ventral gradation of the falciger blade length (dorsal blade 1.5 times longer than ventral one in *Sphaerosyllis levantina* sp. n.) and *Sphaerosyllis sandrae* has smooth falcigerous blades posteriorly and parapodial glands with hyaline material (strongly serrated blades throughout the body and parapodial glands with fibrillar material in *Sphaerosyllis levantina* sp. n.). All the above species differ from *Sphaerosyllis levantina* sp. n. by lacking a subdistal spine on the blades of the falcigers. The only *Sphaerosyllis* species known to possess this spine are *Sphaerosyllis hystrix* Claparède, 1863, *Sphaerosyllis parabulbosa* San Martín and López, 2002 and *Sphaerosyllis boeroi* Musco, Çinar & Giangrande, 2005. *Sphaerosyllis parabulbosa* clearly differs from *Sphaerosyllis levantina* sp. n. by having minute dorsal cirri and antennae, by the presence of a subdistal spine only on blades of the posterior falcigers and by smooth blades of posterior falcigers. *Sphaerosyllis boeroi* differs from *Sphaerosyllis levantina* sp. n. in having much longer blades of the falcigers which show a more pronounced dorso-ventral gradation (dorsal blades 2.6 times longer than ventral ones in *Sphaerosyllis boeroi*, 1.5 times longer in *Sphaerosyllis levantina* sp. n.) than those of *Sphaerosyllis levantina* sp. n. (Figs 3, 5, see also tables in online supplementary material), by having a subdistal spine on blades of all falcigers (only on dorsalmost ones in *Sphaerosyllis levantina* sp. n.) and by the dorsalmost falcigers being serrated only proximally. *Sphaerosyllis hystrix*, according to the literature, has a subdistal spine only on the blades of the anterior dorsalmost falcigers. However, in the examined material of *Sphaerosyllis hystrix* from the Aegean Sea 8 out of 21 specimens also possessed a subdistal spine in posterior falcigers. *Sphaerosyllis hystrix* can nevertheless be distinguished from *Sphaerosyllis levantina* sp. n. by having smooth or finely serrated posterior falcigers (serration less than half the length of the subdistal spine), even when the spine is present (serration almost as long as subdistal spine in *Sphaerosyllis levantina* sp. n.) (Figs 2f, 3, 6). Furthermore, the blades of the dorsalmost falcigers show an anteroposterior gradation in length in *Sphaerosyllis hystrix* (anteriorly 1.5 times longer than posteriorly), whereas they are of similar length throughout the body in *Sphaerosyllis levantina* sp. n. (Figs 3, 6, see also tables in online supplementary material). Finally, *Sphaerosyllis hystrix* has a very narrow pharynx (almost half the width of proventricle), whereas the pharynx of *Sphaerosyllis levantina* sp. n. is wider than ¾ of the width of the proventricle. An identification key to the Mediterranean *Sphaerosyllis* species is provided at the end of this manuscript.

Ben-Eliahu (1977) discusses two different morphological forms of *Sphaerosyllis hystrix* occurring in her samples from Israel. Based on her description and illustrations, the animal identified as *Sphaerosyllis hystrix* sensu Westheide 1974 could potentially belong to *Sphaerosyllis levantina* sp. n. because of the similar characters of falcigers andpapillation. However, the description does not report the characteristic subdistal spine on the blades of the posterior falcigers. In addition, Westheide’s (1974) description of *Sphaerosyllis hystrix* from the Galápagos Islands differs from both Ben-Eliahu’s specimen and the present material by the absence of parapodial glands (Westheide 1974), a character considered as variable and thus of no taxonomic value by Ben-Eliahu (1977) but recently accepted as a taxonomically stable character (Riser 1991).

### Multivariate morphometrical analysis

The results of the Principal Component Analysis show that the first principal component (PC1) account for 77.4% of the variability, the second (PC2) for 16.4% and the remaining 3 PCs for 5.1% (eigenvector values available at http://polychaetes.marbigen.org/content/morphometric-analysis-pca-eigenvectors). The Spearman’s correlation of the Principal Component scores with the measured character values of the individuals revealed that the length of the dorsalmost falcigerous blades in all body parts (anterior, midbody, posterior), as well as the ratio of the anterior to posterior ventralmost falcigerous blade are the most important characters discriminating between the three species (ρ-values >0.8 / <-0.8 at p < 0.005) (http://polychaetes.marbigen.org/content/spearmans-correlation-principal-component-scores-vs-measurements).

**Figure 7.**
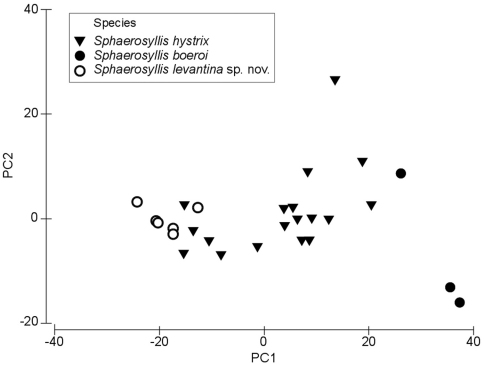
PCA plot.

**Figure 8.**
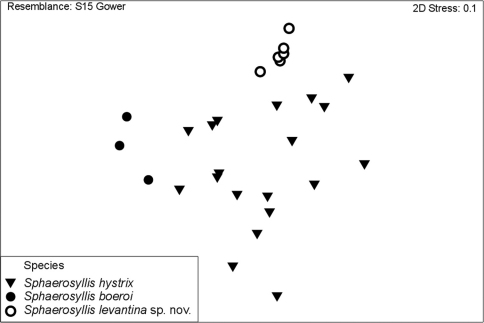
MDS plot.

The PCA plot of the first two components show a discrimination of species into three groups, with individuals of *Sphaerosyllis levantina* sp. n. having the lowest PC1 scores, *Sphaerosyllis boeroi* the highest scores. *Sphaerosyllis levantina* sp. n. and *Sphaerosyllis hystrix* show similar PC2 scores, whereas *Sphaerosyllis boeroi* shows lower scores, and, except for one small-sized individual, forms a distinct group apart from the remaining species. Individuals of *Sphaerosyllis levantina* sp. n. likewise form a close group, however, a couple of individuals of *Sphaerosyllis hystrix* cannot be distinguished from this cluster (Fig. 7). The MDS diagram gives similar results, with individuals of *Sphaerosyllis boeroi* and *Sphaerosyllis levantina* sp. n. forming distinct groups, whereas individuals of *Sphaerosyllis hystrix* are spread as a heterogeneous group, with some of them being plotted close to individuals of either *Sphaerosyllis boeroi* or *Sphaerosyllis levantina* sp. n. (Fig. 8).

The PERMANOVA analysis results in a *p*-value of 0.001 as calculated by 999 permutations, thus the null-hypothesis (no differences between the groups) cannot be sustained. Subsequent analyses of the differences between species through pairwise tests reveals significant differences between species (*Sphaerosyllis hystrix* / *Sphaerosyllis boeroi*: *p* = 0.003, 713 permutations; *Sphaerosyllis hystrix* / *Sphaerosyllis levantina* sp. nov: *p* = 0.001, 995 permutations; *Sphaerosyllis boeroi* / *Sphaerosyllis levantina* sp. n.: *p* = 0.015, 84 permutations).

## Discussion

The genus *Sphaerosyllis* –like many of the small-sized Exogoninae genera– has a difficult and often confused taxonomy and biogeography. Among the potential causes contributing to the current confusion the following could be cited: a) lack of detail in older (before ca. 1970) species descriptions; b) difficulties of observing certain characters in fixed material (Riser 1991); c) descriptions of new species without examination of comparative material; d) ongoing discussions on the taxonomic value of characters such as the presence or absence of dorsal cirri on the second chaetiger (Fauvel 1923, San Martín 2005), presence and type of parapodial glands (Westheide 1974, Ben-Eliahu 1977, Riser 1991) and variations in chaetal structures (Riser 1991). These factors have lead to the assignment of individuals with very different character sets to the same species name and thus to wide-spread distribution records of some species. *Sphaerosyllis hystrix* (type locality Normandy, France) is included among those species with an alleged cosmopolitan distribution, since it has been recorded from most European coasts including the Mediterranean Sea, the north-western coasts of America (Berkeley and Berkeley 1948, Hartman 1968), the Galápagos Islands (Westheide 1974), China (Men et al. 1993, Ding and Westheide 2008), Australia (Hartmann-Schröder 1984, 1985) and the Western Atlantic (Hartman and Fauchald 1971, Temperini 1981), among others. However, recent studies suggest that the North American records of *Sphaerosyllis hystrix* and *Sphaerosyllis pirifera* Claparède, 1868 are in fact individuals of *Sphaerosyllis californiensis* Hartman, 1966 and that the two European species are not represented in the American Pacific fauna (Kudenov and Harris 1995). Similarly, some specimens from the Mediterranean Sea previously identified as *Sphaerosyllis hystrix* had been re-examined and found to exhibit significant morphological differences to *Sphaerosyllis hystrix*, leading to the establishment of a new species, *Sphaerosyllis boeroi* (Musco et al. 2005). In the light of an ever-increasing number of molecular analyses revealing cryptic species complexes in morphologically indistinguishable polychaete species with an assumed cosmopolitan distribution (e.g. Westheide and Hass-Cordes 2001, Westheide and Schmidt 2003, Barroso et al. 2009, Bleidorn et al. 2006) it is likely that the various specimens recorded under the name *Sphaerosyllis hystrix* may in fact form a complex of similar species, especially since many descriptions differ substantially from each other (see Ben-Eliahu 1977).

The morphometric analysis conducted in this study support the hypothesis of several morphologically very similar species co-existing in the Mediterranean. The individuals of *Sphaerosyllis levantina* sp. n. and *Sphaerosyllis boeroi* form distinct groups in the PCA and MDS plots, however the individuals of *Sphaerosyllis hystrix* show a much wider spread, marginally overlapping with the other two species when only the meristic characters are taken into account. This is explained through a high character variability in the examined individuals, especially concerning the presence of a subdistal spine on the blades of the posterior falcigers and the length of the falciger blades. The presence of a subdistal spine on all dorsal falcigerous blades is invariable in *Sphaerosyllis boeroi* and *Sphaerosyllis levantina* sp. n., wheras individuals of *Sphaerosyllis hystrix* with otherwise very similar chaetal structures might or might not possess such spine. Another feature that seems to be highly variable in *Sphaerosyllis hystrix* is the length of the falciger blades in relation to body size. In fact, individuals of *Sphaerosyllis levantina* sp. n. with short falciger blades are located at the lower end of the size spectrum of all measured blades, *Sphaerosyllis boeroi* with almost spiniger-like blades at the higher end, whereas the blade lengths of the examined individuals of *Sphaerosyllis hystrix* form a smooth transition between the other two species.

However, when tested by strict statistical criteria, the hypothesis of different co-existing species is significantly supported, and based on their meristic characters the species show significant differences. The results of the current study suggest that *Sphaerosyllis hystrix* may well constitute a species complex. Given the difficult taxonomic status of the genus, similar results might be expected for other species as well, and consequently, distributions of several *Sphaerosyllis* species might be in fact questionable or unknown.

### Key to the Mediterranean Sphaerosyllis species:

The three species *Sphaerosyllis claparedei* Ehlers, 1864, *Sphaerosyllis papillifera* Naville, 1933 and *Sphaerosyllis ovigera* Langerhans, 1879 are poorly known. All have been described as having dorsal cirri on the second chaetiger, however, other species, such as *Sphaerosyllis hystrix*, were also originally described or illustrated with dorsal cirri on the second chaetiger whereas they are in fact absent. Since the three aforementioned species are exclusively known from their original description (or partly reproductions of these) and have never been re-described based on new material, they are tentatively included in the key below, but their identity remains questionable.

**Table:** 

1	Dorsal cirri on chaetiger 2 present	2
–	Dorsal cirri on chaetiger 2 absent	4
2	Papillae on dorsum absent	*Sphaerosyllis claparedei* Ehlers, 1864
–	Papillae on dorsum present	3
3	Parapodial glands absent	*Sphaerosyllis papillifera* Naville, 1933
–	Parapodial glands with fibrillar material	*Sphaerosyllis ovigera* Langerhans, 1879
4	Parapodial glands present	5
–	Parapodial glands absent	15
5	Parapodial glands with fibrillar material	6
–	Parapodial glands with granular material	12
6	All antennae in line	*Sphaerosyllis capensis* Day, 1953
–	Median antenna inserted more posteriorly than lateral ones	7
7	Dorsal cirri shorter than parapodial lobes, at least in anterior chaetigers	8
–	Dorsal cirri longer than parapodial lobes	9
8	Blades of falcigers strongly serrated, short (<10µm); shafts with strong spines	*Sphaerosyllis thomasi* San Martín, 1984
–	Blades of falcigers with serration only anteriorly and dorsalmost; blades with slight dorso-ventral gradation but always longer than 10µm; shafts smooth	*Sphaerosyllis parabulbosa* San Martín and López, 2002
9	Blades of falcigers without marked dorso-ventral gradation in length	*Sphaerosyllis taylori* San Martín, 1984
–	Blades of dorsalmost falcigers at least 1.5 times the length of ventral ones	10
10	Blades of posterior dorsal compound falcigers smooth to finely serrated	*Sphaerosyllis hystrix* Claparède, 1863
–	Blades of posterior dorsal compound falcigers strongly serrated (spinules of almost same length as the subdistal spine)	11
11	Blades of anterior dorsal compound falcigers at least twice as long as ventral ones; anteroposterior gradation of blade length; blades of both dorsal and ventral compound chaetae with a subdistal spine	*Sphaerosyllis boeroi* Musco, Çinar and Giangrande, 2005
–	Blades of anterior dorsal compound falcigers less than twice as long as ventral ones; no anteroposterior gradation of blade length; blades of only dorsal compound chaetae with a subdistal spine	*Sphaerosyllis levantina* sp. n.
12	Blades of dorsalmost falcigers long (>30µm), at least twice as long as ventral ones	*Sphaerosyllis magnidentata* Perkins, 1981
–	Blades of falcigers short (<15µm), with only slight dorso-ventral gradation	13
13	Dorsal cirri clearly longer than parapodial lobes	*Sphaerosyllis* sp. [San Martín, 2003]
–	Dorsal cirri as long as or shorter than parapodial lobe	14
14	Antennae bulbous with small tip, shorter than prostomium; dorsal simple chaetae smooth; anterior parapodia with two aciculae, one straight, one with tip bent at right angle	*Sphaerosyllis* sp. Del-Pilar-Ruso & San Martín, in press
–	Antennae pyriform, as long as prostomium, dorsal simple chaetae serrated, all parapodia with one acicula	*Sphaerosyllis glandulata* Perkins, 1981
15	All aciculae straight	16
–	Tip of some aciculae bent at right angle	17
16	Dorsal cirri with conspicious papilla, giving cirri a bifid appearance	*Sphaerosyllis gravinae* Somaschini and San Martín, 1994
–	Doral cirri without papilla	*Sphaerosyllis bulbosa* Southern, 1914
17	All antennae in line	*Sphaerosyllis austriaca* Banse, 1959
–	Median antenna inserted more posteriorly than lateral ones	18
18	Anterior parapodia with two aciculae, one straight, one with tip bent at right angle; pharyngeal glands on chaetiger 1 present	*Sphaerosyllis pirifera* Claparède, 1868
–	All parapodia with one acicula only; pharyngeal glands on chaetiger 1 absent	*Sphaerosyllis piriferopsis* Perkins, 1981

## Supplementary Material

XML Treatment for Sphaerosyllis levantina

## Supplementary material

The Scratchpads version of this publication is available at:

http://polychaetes.marbigen.org/node/35

Character matrices for specimens used in the morphological analysis are available at:

http://polychaetes.marbigen.org/content/measured-values-sphaerosyllis-specimens

Summary statistics for the species are available at:

http://polychaetes.marbigen.org/content/summary-statistics-sphaerosyllis-hystrix

http://polychaetes.marbigen.org/content/summary-statistics-sphaerosyllis-boeroi

http://polychaetes.marbigen.org/content/summary-statistics-sphaerosyllis-levantina

Results of the morphometric analysis are available at:

http://polychaetes.marbigen.org/content/morphometric-analysis-pca-eigenvectors

http://polychaetes.marbigen.org/content/spearmans-correlation-principal-component-scores-vs-measurements

Illustrations and graphs of the statistical analysis are available at:

http://polychaetes.marbigen.org/category/image-galleries/sphaerosyllis
